# Application of Cyanobacteria (*Roholtiella* sp.) Liquid Extract for the Alleviation of Salt Stress in Bell Pepper (*Capsicum annuum* L.) Plants Grown in a Soilless System

**DOI:** 10.3390/plants11010104

**Published:** 2021-12-30

**Authors:** Adewale Suraj Bello, Radhouane Ben-Hamadou, Helmi Hamdi, Imen Saadaoui, Talaat Ahmed

**Affiliations:** 1 Department of Biological and Environmental Sciences, College of Arts and Sciences, Qatar University, Doha P.O. Box 2713, Qatar; a.suraj@qu.edu.qa (A.S.B.); benhamadou@qu.edu.qa (R.B.-H.); 2 Center for Sustainable Development, Qatar University, Doha P.O. Box 2713, Qatar; hhamdi@qu.edu.qa (H.H.); imen.saadaoui@qu.edu.qa (I.S.); 3 Environmental Science Centre, Qatar University, Doha P.O. Box 2713, Qatar

**Keywords:** *Roholtiella* spp., salinity stress, Bell pepper (*Capsicum annuum* L.), foliar spray/application

## Abstract

Salinity is one of the abiotic stresses that affect crop growth and productivity in arid and semi-arid regions. Unfortunately, there are few known methods to mitigate the deleterious impacts of salt stress on the development and yield of vegetable crops. Blue-green algae (cyanobacteria) are endowed with the potential to curb the negative impacts of salt stress as they are characterized by biostimulant properties. The present work aimed to investigate the effects of *Roholtiella* sp. as a foliar extract on the growth characteristics, physiological and biochemical responses of bell pepper (*Capsicum annuum* L.) plants under varying levels of salinity conditions. A soilless water experiment was carried out in a greenhouse where bell pepper seedlings were grown under five salt concentrations (0, 50, 200, 150, and 200 mM of NaCl). Growth characteristics, pigments content, relative water content, and antioxidant activity (CAT) were determined. Our results showed that growth parameters, relative water content (RWC), chlorophyll a & b concentrations under salinity conditions were negatively affected at the highest concentration (200 mM). Interestingly, the application of *Roholtiella* sp. foliar extract enhanced the plant growth characteristics as shoot length increased by 17.014%, fresh weight by 39.15%, dry and weight by 31.02%, at various salt treatments. Moreover, chlorophyll a and b increased significantly compared with seedlings sprayed with water. Similarly, RWC exhibited a significant increase (92.05%) compared with plants sprayed with water. In addition, antioxidants activities and accumulation of proline were improved in *Roholtella* sp. extract foliar sprayed seedlings compared to the plants foliar sprayed with water. Conclusively, at the expiration of our study, the *Rohotiella* sp. extract-treated plants were found to be more efficient in mitigating the deleterious effects caused by the salinity conditions which is an indication of an enhancement potential of tolerating salt-stressed plants when compared to the control group.

## 1. Introduction

Several abiotic stresses viz drought (water stress), excessive water accumulation (waterlogging), extreme temperatures (cold, frost, and heat), and salinity, etc. are often responsible for poor crop production [1]. Thus, salinity is one of the serious and increasing challenges mitigating optimum crop production particularly the quality and quantity (yield) of the crop in arid and semi-arid countries [2,3]. Globally, over 9.0 × 10^2^ million hectares of land are affected by salination, which constitutes one-fifth of total cultivable land [1,4]. Consequently, there is an urgent need for proactive measures to improve plant productivity and crop output under salination conditions to solve the problem of increasing food demand of the world population [5,6,7,8]. The incidence of abiotic stress has been increasing lately because of global warming leading to the persistent rise in unfavorable weather conditions [9]. However, several studies have established a deleterious effect of the increasing rate of soil salinity on crop productivity globally [10,11]. Accumulation of salt may lead to an alteration in physiological, molecular, metabolic activities, nutrient deficiency, ion toxicity, and water deficit or water potential reduction [2,10,11,12,13,14]. Also, in other studies, it has been demonstrated that the impact of toxicity due to higher concentration of salt in soil may be regulated or conciliated through the production of antioxidants as well as hampering or obstructing the generation of reactive oxygen species (ROS) [14,15]. Naturally, the sensitivity of plants to salination is general and predominant in respective of the type of plant/crop simply because there is a likelihood of disruption in the interaction of the natural microbes in soil and inhibit the growth of microbial in their natural ecosystem [6,16].

Microalgae, viz. cyanobacteria, and eukaryotic algae can manufacture their foods using radiant energy, thus they are photoautotrophic microorganisms. The application of microalgae as biostimulants and biofertilizers is gaining popularity as a potential and sustainable alternative to the inorganic fertilizer with a wider acceptance by farmers and agrochemical industries [17]. Interestingly, several studies have established that microalgae exhibits biostimulants properties that enhance the resistance of crops to the deleterious impacts of abiotic stress mostly salt stress [1,18]. Similarly, wastewater may be used as a source of raw material in microalgae production because of its richness in organic nutrients, consequently, minimizing the usual huge cost of production [3,19]. Naturally, algae extracts may have a positive impact to alleviate abiotic stresses in plants by concentrating/targeting different pathways [20].

Bell pepper (*Capsicum annuum* L.) is an essential vegetable crop because of its economic value and health benefits, thus, it is cultivated across all the continents of the world. Bell pepper (*Capsicum annuum* L.) fruits contain ascorbic acid as well as lycopene, a worthy or treasured compound containing anti-oxidant and anti-cancer characteristics. Thus, its cultivation, usefulness, and consumption are rising every year [21,22]. However, bell pepper is believed to have an adaptable potential to harsh climatic change; still, rising soil and water salination hinder the development, output, and fruit quality of fruit, thus leading to a huge reduction in productivity [23,24,25]. In an attempt to mitigate the negative effects engendered by salinity stresses, researchers have undertaken different measures such as using marine resources such as macro and microalgae as elicitors to enhance crop production [26].

This study is an advancement on the previous study [17] conducted to screen three unknown cyanobacteria strains in which the most effective strain *Roholtiella* sp. was selected to further investigate its potential benefit as a stress alleviator. However, up till now, little research has been conducted to describe the impact of the application of cyanobacteria extract on plant performance, productivity, and salt stress alleviation, thus, to the best of our understanding, this study was never carried out before in bell pepper (*Capsicum annuum* L.) under a salt-affected soilless system using *Roholtiella* spp. strain. Therefore, the novelty of this investigation was to establish the positive impact of *Roholtiella* spp. extract (foliar application/spraying) alleviation of bell pepper plant under salinity stress. Nonetheless, the significant discovery/result could be extended to other crops as a model and for agricultural farming.

## 2. Results

### 2.1. Cyanobacteria Nutrient Composition

Nutrient composition of Roholtiella sp. extract in part per million (ppm) contained a considerable amount of Sodium (Na^+^) 2.379, Ammonium (NH_4_^+^) 0.674, Potassium (K^+^) 8.533, Calcium (Ca^2+^) 1.777, Magnesium (Mg^2+^) 3.483, Fluoride (F^−^) 0.0191, Chloride (Cl^−^) 3.168, Nitrate (NO_3_^−^) 5.247, Phosphate (PO_4_^3−^) 13.67, Sulphate (SO_4_^2−^) 0.212. In a like manner, the spectra scan of this strain showed that it contains a reasonable amount of phycoerythrin and phycocyanin [17].

### 2.2. Effect of Salt Stress and Foliar Spray on Vegetative Parameters

**Figure 1:**
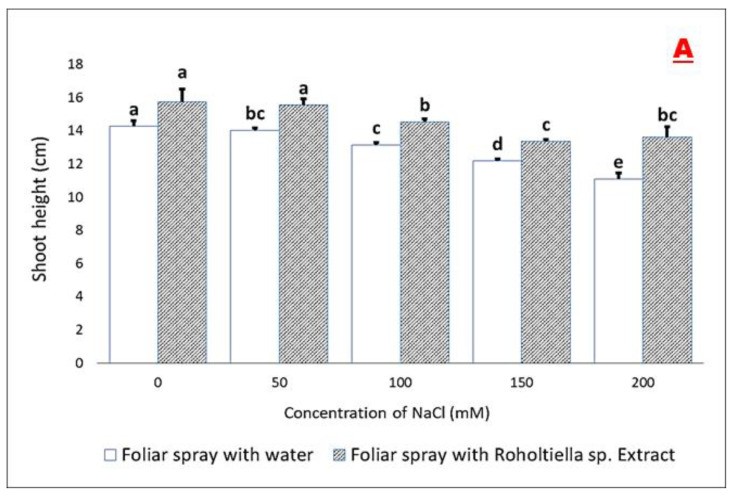

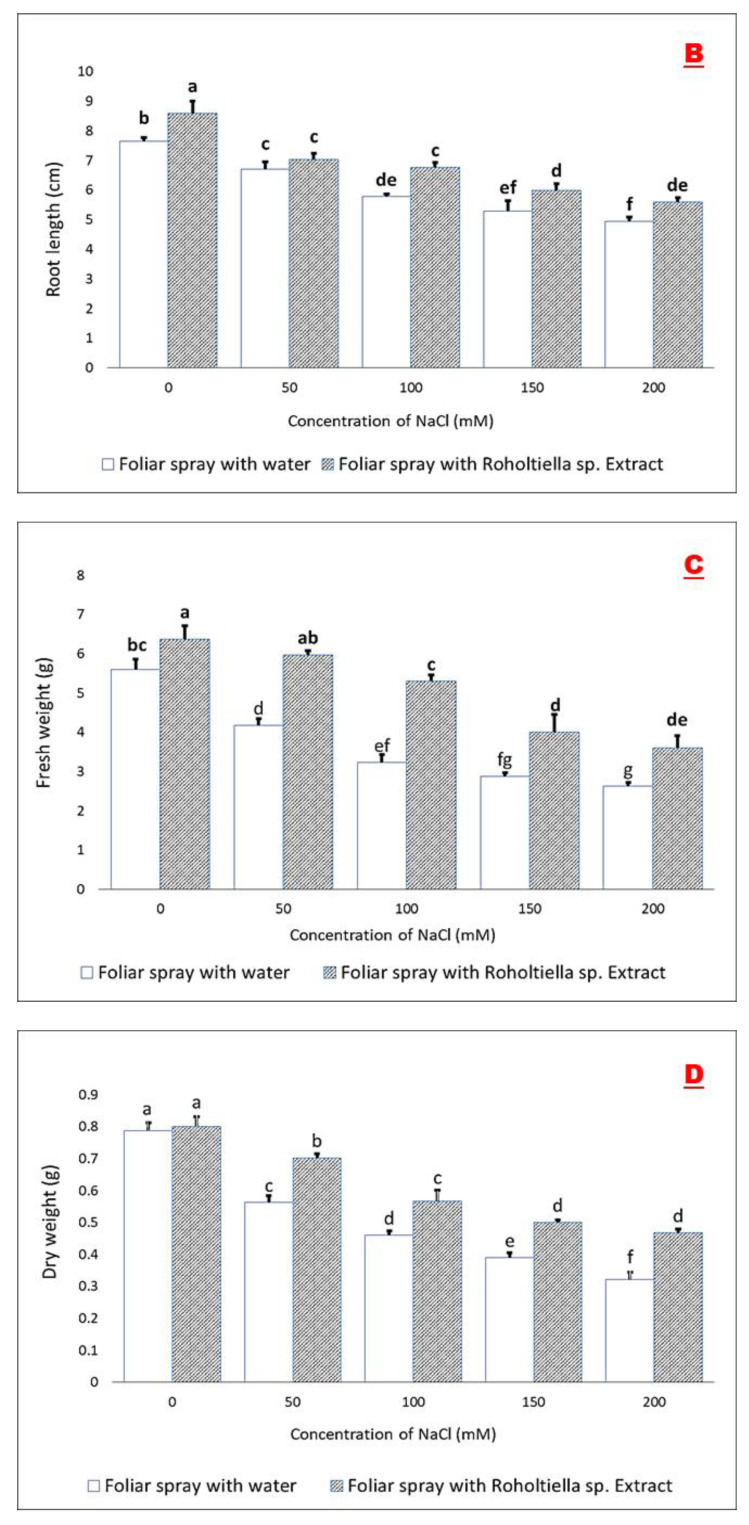

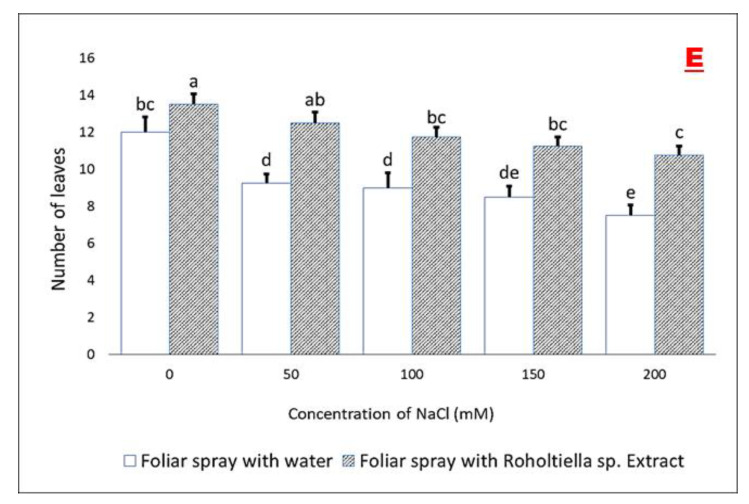
Effect of *Roholtiella* sp. extract on the shoot height (**A**), root length (**B**), fresh weight (**C**), dry weight (**D**), and (**E**) the number of leaves in salt-stressed bell pepper seedlings. The different letters (a–g) indicate significantly different values in response to the different treatments.

The obtained results as shown in Figure 1A–E respectively indicated that salt-stressed bell pepper plants exposed to various concentration levels caused a significant decrease in the vegetative parameters measured which are shoot length, root length, fresh weight, dry weight, and the number of leaves plant^−1^. The shoot length was significantly influenced by the *Roholtellia* sp. extract and the degree of salt concentration level. At the 0 mM salt concentration, there was no significant difference between the control (0 Mm) and foliar treated plants though the shoot length increased by (9.33%) (Figure 1A). Also, at the 50, 100, 150, and 200 mM salt concentrations and compared with those plants’ foliar sprayed with water, the shoot height increased by 9.77%, 8.68%, 8.28%, and 17.04% respectively. Also, the trend is similar with the root length as there was a significant difference at all the salt treatment levels 0–200 mM. The seedling sprayed with *Roholtiella* sp. exhibited a significant increase in root length by 11.05%, 4.63%, 14.76%, 11.67%, and 11.61% compared with the control group sprayed with water. In addition, there was a significant increase in the fresh weight, dry weight, and the number of leaves of the treated stressed seedlings (0–200 mM) with *Roholtiella* sp. compared with the control group that was treated with water. For the plant fresh weight, it was significantly increased by 12.16%, 30.13%, 39.15%, 28.13%, and 27.08%, while for the plant dry weight, the pattern is the same as it significantly increased except at 0 mM by 1.56%, 19.93%, 18.94% 22% and 31.02%. The number of leaves of the stressed plant (0–200 mM) significantly increased by 11.11% 26% 23.4% 24.44% and 30.23% respectively.

### 2.3. Effect of Roholtiella sp. Extract on Relative Water Content (RWC)

**Figure 2:**
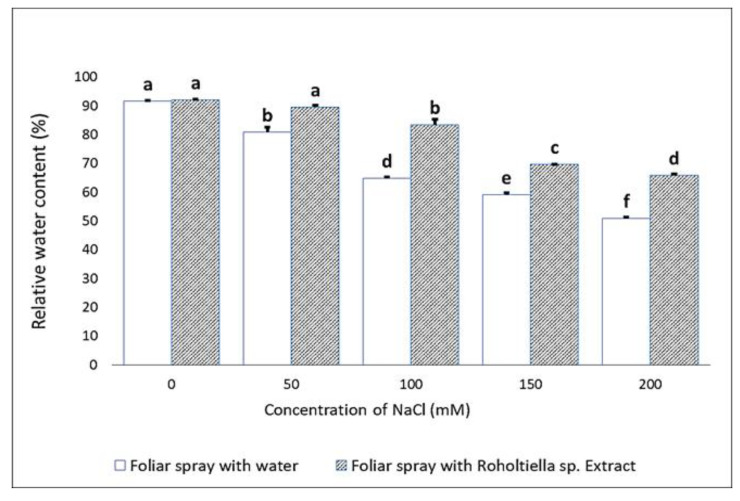
Effect of *Roholtiella* sp. extract on the relative water content of leaves in salt-stressed bell pepper seedlings. The different letters (a–f) indicate significantly different values in response to the different treatments.

The obtained results in Figure 2 showed relative water content (RWC) of the stressed bell pepper plants reduced with an increased level of salinity stress (0–200 mM). The seedlings foliar sprayed with water showed 91.58%, 80.76%, 64.78% 59.07%, and 50.87% in RWC. In a like manner, the seedlings foliar sprayed with *Roholtiella* sp. extract showed 92.05%, 89.51% 83.39% 69.61% and 65.77% in RWC. Thus, it shows that the seedlings foliar sprayed with *Roholtiella* sp. extract exhibited a significant increase in RWC when compared with the seedlings foliar sprayed with water and their respective control (0 mM) groups.

### 2.4. Effect of Salt Stress and Foliar Spray on Chlorophyll Pigments and Proline Concentration

**Figure 3:**
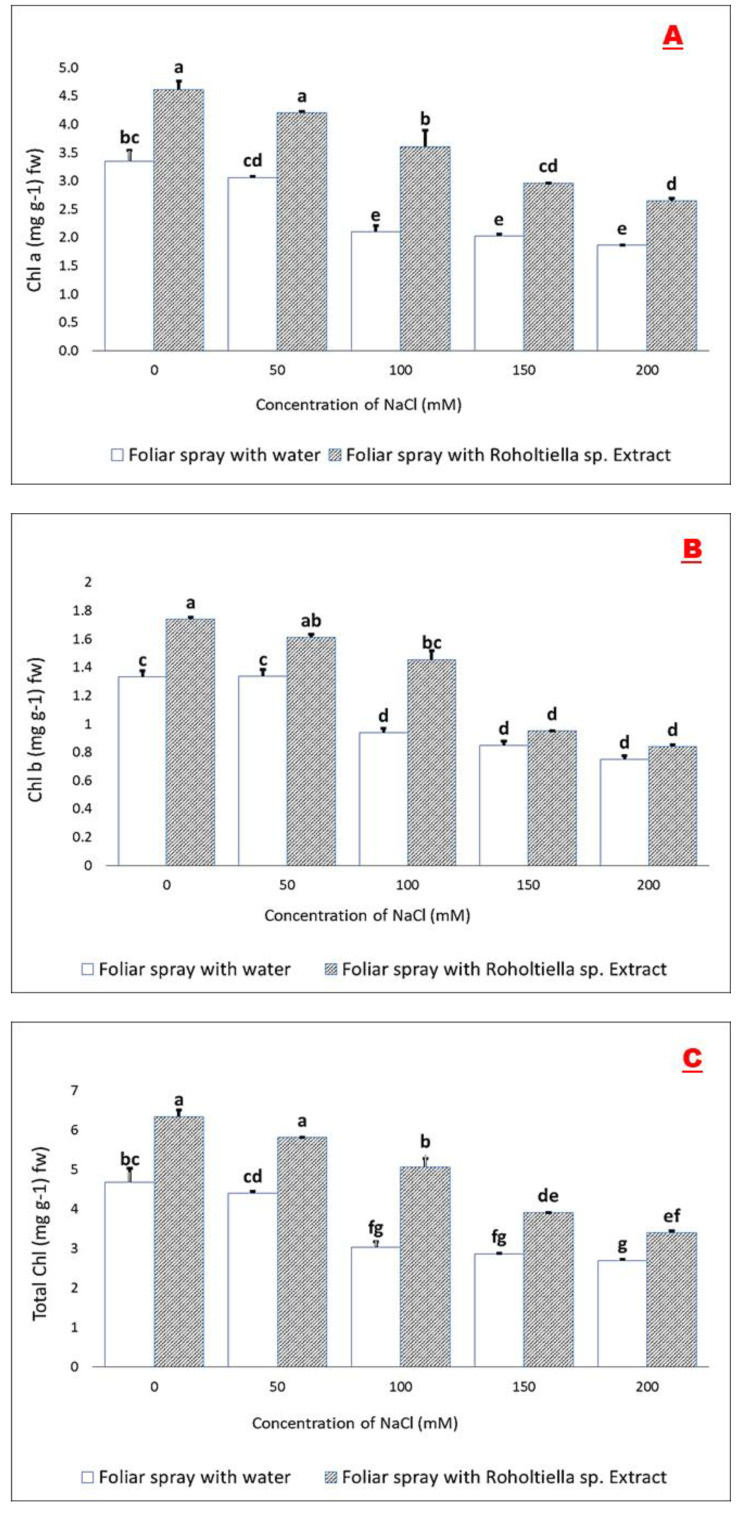

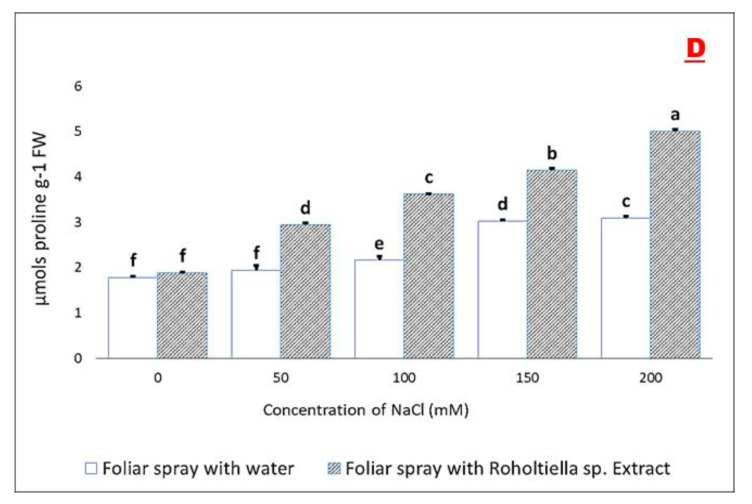
Effect of *Roholtiella* sp. extract on contents of chlorophyll-a (**A**), chlorophyll b (**B**), total chlorophyll (**C**), and proline (**D**) in bell pepper seedlings under salt stress. Bars accompanied with different letters indicate significant differences according to Turkey’s test at a significant level of 5% (*p* < 0.05). Salinity concentrations are 0, 50, 100, 150, and 200 mM. The different letters (a–f) indicate significantly different values in response to the different treatments.

It is clear from the obtained results in Figure 3 that there was a significant change in the content of the pigment on the foliar treated plants of bell pepper with *Roholtiella* sp. extract compared with the group treated with water. The concentration of chlorophyll-a (3.34, 3.06, 2.1, 2.02, and 1.86 mg g^−1^ Fw), chlorophyll b (1.33, 1.34, 0.94, 0.85, and 0.75 mg g^−1^ Fw), and total chlorophyll (4.68, 4.4, 3.04, 2.87, and 2.7 mg g^−1^ Fw) significantly declined in plants of bell pepper under all concentrations of salinity (0, 50, 100, 150, and 200 mM) respectively.

Furthermore, the application of *Roholtiella* sp. extract lead to a significant increase of chlorophyll-a (4.61, 4.20, 3.60, 2.96, and 2.65 mg g^−1^ Fw) when compared with the those plants foliar sprayed with water (3.49, 3.13, 2.36, 2.23, and 1.82 mg g^−1^ FW) at 0, 50, 100, 150, and 200 mM salinity level respectively. In a like manner, chlorophyll b (1.74, 1.61, 1.46, 0.95, and 0.84 mg g^−1^ Fw) and total chlorophyll (6.35, 5.81, 5.06, 3.91, and 3.40 mg g^−1^ FW) increased significantly when treated with the extract compared to the untreated seedlings (1.33, 1.34, 0.94, 0.85, and 0.75 mg g^−1^ Fw) and (4.68, 4.4, 3.04, 2.87, and 2.7 mg g^−1^ FW) at all the concentration levels (0, 50, 100, 150, and 200 mM) of salt stress. However, even at zero salinity level, there was an incremental in the chlorophyll content level because of the growth enhancer potential of the *Rholtiella* sp. extract [17]. Generally, the proline accumulation increases faster in stressed plants at different conditions when compared with other amino acids [26]. The bell pepper seedlings sprayed with Roholtiella sp. extract exhibited an increase in the proline accumulation at all the concentration levels of NaCl as compared to those treated with water at the same NaCl level. Thus, proline concentrations (1.79, 1.95, 2.16, 3.03, 3.10µmols proline g^−1^ FW) under salinity concentrations (0, 50, 100, 150, and 200 mM) significantly increased in bell pepper seedlings compared to the group treated with water. However, the accumulation of proline increases under the application of *Roholtiella* sp. to 1.88, 2.94, 3.63, 4.15, 5.01 µmols proline g^−1^ FW corresponding to salt-stressed seedlings at 0, 50, 100, 150, and 200 mM respectively. Thus, Bell pepper (*Capsicum annuum* L.) plants treated with the *Roholtiella* sp. extract showed significant impacts on the content of proline under the control (0 mM) and all the salinity levels (50, 100, 150, and 200 mM). However, the observed significant percentage increase recorded was 5.18%, 33.89%, 40.32%, 26.96%, and 38.20% in the plants treated with extract compared to those foliar sprayed with water at all the salinity levels.

### 2.5. Effect of Salt Stress and Foliar Spray on the Antioxidant Capacity and Activity

**Figure 4:**
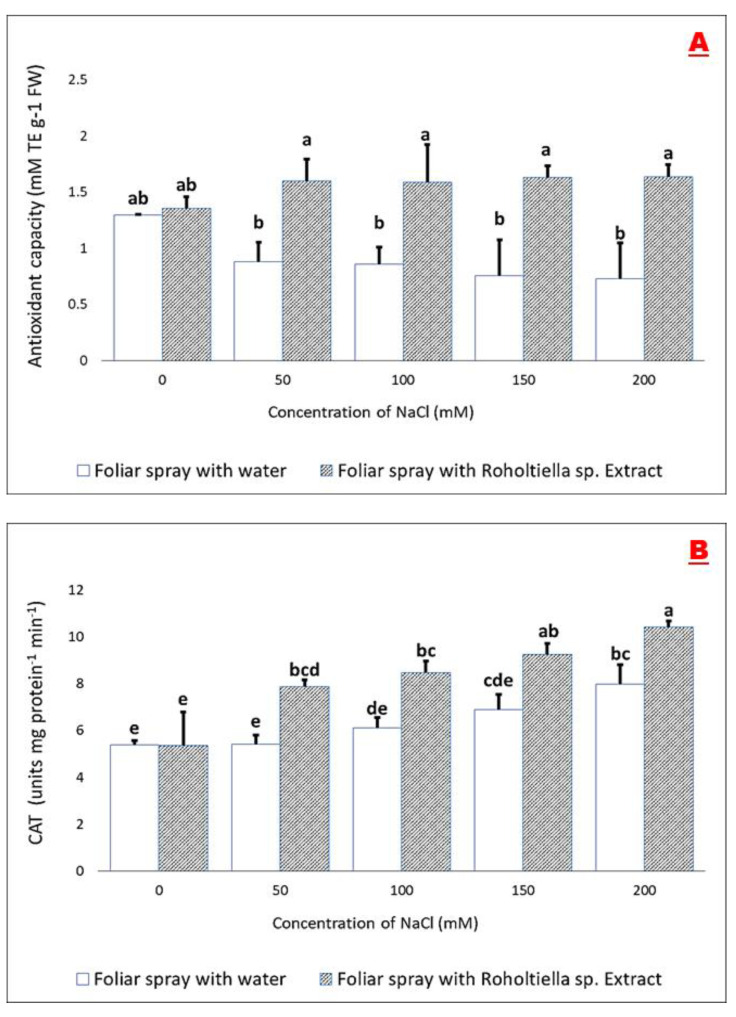
Effect of *Roholtiella* sp. extract on the Trolox equivalent antioxidant capacity (**A**) and activity of catalase (CAT) (**B**) in induced bell pepper seedlings under salt stress. Bars accompanied with different letters indicate significant differences according to Turkey’s test at a significant level of 5% (*p* < 0.05). Salinity concentrations are 0, 50, 100, 150, and 200 mM. The different letters (a–d) indicate significantly different values in response to the different treatments.

ABTS assay is one of the most available procedures commonly used for evaluating the antioxidant capacity (ABTS^+^ radical scavenging capacity) of plants [27]. The recorded values of ABTS^+^ free radical scavenging potential/ability in the induced plants foliar sprayed with water and *Roholtiella* sp. extract exhibited significant differences Figure 4A. In the ABTS procedures, the antioxidant capacity of bell pepper treated with water in control (0 mM) plants was (1.3 mM TE g^−1^ FW). However, in responding to salinity, antioxidant capacity decreases as salinity increases, the highest value (0.88 mM TE g^−1^ FW) was recorded at 50 mM and further reduced significantly (0.86 mM TE g^−1^ FW) at 100 mM. At 150 and 200 mM of salt concentrations, the seedlings exhibited more weakness in antioxidant capacity at 0.76 mM TE g^−1^ FW and 0.73 mM TE g^−1^ FW respectively. Interestingly, when the plants were sprayed with extract, among all the concentrations, 200 mM is the highest, attaining 1.64 mM TE g^−1^ FW followed by 150 mM (1.63 mM TE g^−1^ FW), 100, 50 Mm reaching (1.6 mM TE g^−1^ FW and 1.59 mM TE g^−1^ FW) respectively when compared with the group sprayed with water but there is no significant difference at the concentration of 0 mM (1.36 mM TE g^−1^ FW).

In a like manner, the obtained results as shown in Figure 4B indicate Bell pepper plants exposed to salt stress at four concentrations (50, 100, 150, and 200 mM) caused a significant increase in the antioxidant enzymatic activity. Catalase activity significantly increased in the stressed bell pepper plants (5.37, 7.87, 8.48, 9.26, 10.42 unit mg protein^−1^ min^−1^) foliar sprayed with *Roholtiella* sp. extract as compared to salt-stressed and water foliar sprayed bell pepper seedlings (5.39, 5.41, 6.12, 6.89, 7.99 unit mg protein^−1^ min^−1^) as well as the control (0 Mm) group. Interestingly, as the concentration levels of NaCl gradually increase, the corresponding catalase activity also increases as observed in Figure 3.

## 3. Discussion

Plant production has been estimated to be lower by as much as 50% simply because of the adverse effect of abiotic stresses on plants [9]. Generally, plants expend most of their energy on essential processes for maintenance, cell rejuvenation, vegetative, and generative growth when grown under non-stressed conditions [3]. Nevertheless, once plants are stressed, resources allocation is affected as more of it is required as salinity increases to reduce the purported stress [2]. Bell pepper (*Capsicum annuum* L.) exhibited reasonable tolerance to salinity conditions when treated with *Roholtiella* sp. extract compared with water-treated stressed seedlings. However, it has been reported that abiotic stress negatively affects plant growth and development, therefore, salinity stress retarding/curbing plant growth and development [28]. Consequently, by definition, salt/salinity tolerance is the ability of a stressed plant to withstand the deleterious impacts of excessive concentration of salt or increasing salinity without recording any significant adverse impacts viz. growth retardation, output reduction, or foliar salt destruction [29]. Interestingly, the positive impacts of microalgae on flora growth and development have been reported by several studies but not much attention has been given to important vegetables such as bell pepper despite its both economic and health benefits. Also, in this study, the evaluated vegetative parameters (shoot height, number of leaves, dry weight, fresh weight, and the number of leaves) are significantly affected as salinity levels increase (Figure 1A–E). High salinity concentration caused the decline in the growth parameter as a result of alteration in the bioprocesses of the plant. However, these growth parameters were found to increase in the seedlings treated/foliar sprayed with *Roholtiella* sp. extract compared to the control group. The shoot length, root length, and the number of leaves significantly increased compared to the control. This result complies with the report from Plaza et al., 2018 study, in which he reported a positive effect from the application of A. *platensis* extract by improving the number of flowers per plant as well as the root dry contents [30]. Similarly, the increase in the shoot length complies with the report from the study conducted by Mansori et al. [31], in which *phaseolus* plant treated with extract exhibited an increase in the shoot and root length respectively. Generally, from our study, the application of *Roholtiella* sp. extract to treat the salt-stressed plants of bell pepper has shown an improvement in their growth performance throughout the experimental period. Furthermore, from our results, it was shown that salinity stress has damaging effects on bell pepper at the concentrations level. The process of photosynthesis greatly depends on chlorophyll a, b, and to some extent the total chlorophyll, a biochemical process that is executed by two reactions which are light and dark sensitive. The first reaction, which is light sensitive, there is the formation of Nicotinamide adenine dinucleotide phosphate (NADPH) and adenosine triphosphate (ATP), while the second reaction is dark sensitive leading to fixing carbon dioxide [28,32]. However, from our results, it is obvious that chlorophyll content decreased significantly under the four salinity concentrations; thus the decremental trend is from the lower concentration (50 mM) to the higher concentration (200 mM) respectively that is likely to be caused by the deleterious effect of salinity stress on the composition of stomata [33,34], because it is a known fact that leaf growth rate retardation and subsequent leaf area reduction are the number one plant responses to salt stress. The cumulative effect of damaging chloroplast structure will be subsequently felt on the energy transportation from PSII to PSI as it decreases [35]. Interestingly, the results obtained are in accordance with previous reports on the cultivation of bell pepper under salinity stress by Abdelaal et al. [25]. Also, according to Asrar et al. 2017, it was previously reported that high salinity concentration leads to deleterious effects on PSII, thus triggering the chloroplast proteins reduction and the concentration of chlorophyll [36]. No doubt, there is a positive relationship between the chlorophyll reduction and the relative water content (RWC) reduction as these are influenced by the varying degree of salinity. However, the foliar sprayed of *Roholtiella* sp. extract reduces the negative effects of salt stress on the chlorophyll content that subsequently enhanced bell pepper growth and rapid development of plants exposed to stressful environments, which conform with an investigation conducted on bell pepper by Al-Kahtani et al. [28]. In addition, the application of *Rohpltiella* sp. extract increasing the chlorophyll content may be connected with the availability of amino acid in most blue-green algae which is equally peculiar with cyanobacteria such as *Roholtiella* sp. as well. These findings comply with a previous study conducted by Possingham [37]. Also, the significant increase in bell pepper plant growth and pigments content in *Roholtiella* sp. extract in the foliar treatment of the stressed seedlings improvement can be connected to the osmotic adjustment as previously reported by Mutale-joan et al. [38]. Generally, the proline accumulation increases faster in stressed plants at different conditions when compared with other amino acids [26]. The bell pepper plants sprayed with Roholtiella sp. extract exhibited an increase in the proline accumulation at all the concentration levels of NaCl as compared to those treated with water at the same NaCl levels. Plants, being immobile or sessile organism that is often affected by different stresses at every growth stage. Proline like every other inert solute or inactive metabolites such as glycine-betaine are regarded as osmolytes because of their potential to suppress osmotic stresses emanated from salt stress in particular. Many studies established that proline contributes essentially to the adjustment of osmotic stresses by indemnifying for the osmotic pressure of cations such as Na^+^ [39,40,41]. From the present study, the accumulation of proline was significantly activated by *Roholtiella* sp. extract in bell pepper seedlings that were put through different NaCl levels (50, 100, 150, and 200 mM) when compared with seedlings sprayed with water. This result correlates with a similar study conducted by Chanda Mutale-joan et al. that exhibited a significant increase in the proline accumulation treated with microalgae extract when compared with the control [38]. In a like manner, *Roholtiella* sp. extract increased reactive oxygen species ROS scavenging enzymatic processes of catalase compared to bell pepper sprayed with water as shown in Figure 3. Tukan in his study expressed that ROS serve as signaling molecules that control bioactivities and ways plant deals with different stresses both the biotic and abiotic [42]. At different salinity conditions, the excessive accumulation of ROS is triggered resulting in a situation capable of destroying the cellular structures of plants [43,44,45]. However, to counter the possible oxidative stress resulting from excessive accumulation of ROS; thus, RSO scavenging mechanism is triggered by plants [46]. Interestingly, in the present work, the activities of CAT in *Roholtiella* sp. Extract-sprayed plants have increased significantly compared to water-sprayed plants. This is an indication that the enhanced activity of CAT in the extract-sprayed plants can scavenge ROS by decomposing H_2_O_2_ into H_2_O and O_2_. This result is in line with several studies that reported that the activities of antioxidant enzymes viz. SOD, POX, and CAT reduce the probable oxidative damage through the ROS decomposition to H_2_O_2_ or by detoxification [44,47,48]. In a like manner, this result is supported by a report from the study conducted by El-Sharkawy et al. detailing the detoxification potential of CAT activity in two different cultivars of Alfalfa that are salt sensitive and tolerance [49]. Similarly, there is a correlation between this result and that of Mittova et al. reported the enhancement of CAT activity mitigating salinity stress in Lycopersicon pennellii, a wild salt-tolerant tomato cultivar [50]. In a like manner, the comprehensive analysis of results of antioxidant capacity of bell pepper plants subjected to salinity at various concentrations obtained from foliar spraying with water and *Roholtiella* sp. extract exhibited that the extract treatment significantly enhanced antioxidant capacity compared with the plant group treated with water.

## 4. Materials and Methods

### 4.1. Plant Material, Experiment Preparation of the Soilless System, and Salt Stress Application

**Figure 5:**
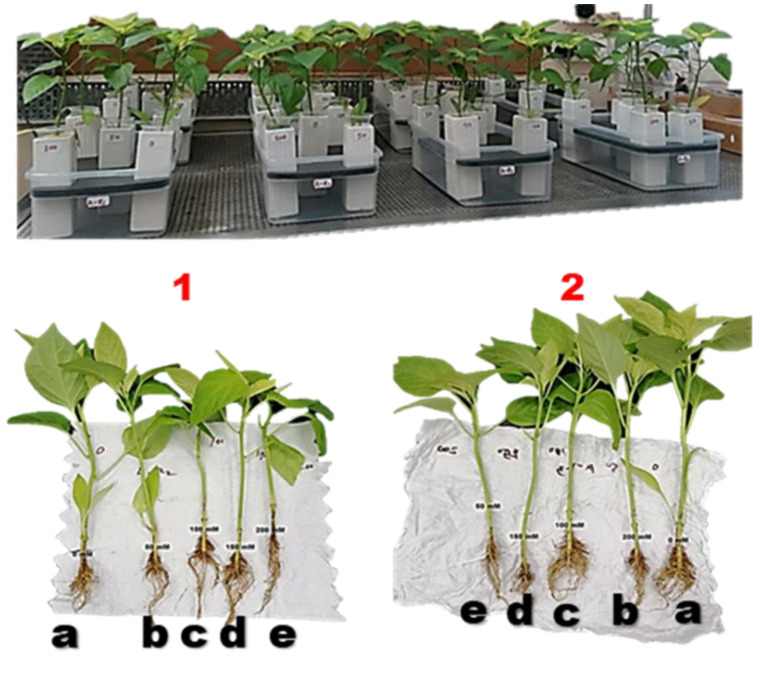
The experimental design and morphological variation of induced NaCl bell pepper plants **1**. Water sprayed, and **2**. *Roholtiella* sp. extract sprayed induced plants after 10 days a: 0 Mm induced plant, b: 50 mM NaCl-induced plants, c: 100 mM NaCl-induced plants, d: 150 mM NaCl-induced plants, and e: 200 mM NaCl-induced plants.

The hybrid bell pepper (*Capsicum annuum* L.) was procured from the certified commercial seed supplier Technical Agricultural Company, Doha, Qatar. Surface sterilization of the seeds was facilitated with 5% NaClO (Sodium hypochlorite) for 5 min and subsequently thoroughly rinsed with deionized water. However, the seeds were first germinated in the germination boxes inside the greenhouse at the Department of Biological and Environmental, Qatar University. After 30 days, healthy seedlings were transferred into a 192 mL glass vase containing Hoagland solution as media in a deep-water culture system hydroponically (soilless culture) Figure 5.

Standard nutrient solution with concentration in g L^−1^ was prepared from Ca(NO_3_)_2_·4H_2_O, 1.250 g L^−1^; KNO_3_, 0.410 g L^−1^; NH_4_H_2_PO_4_, 0.280 g L^−1^; MgCl_2_·6H_2_O, 0.624 g L^−1^; FeSO_4_·7H_2_O, 0.060 g L^−1^; EDTA-Na_2_, 0.080 g L^−1^; H_3_BO_3_, 0.006 g L^−1^; MnCl_2_·4H_2_O, 0.04 g L^−1^; ZnSO_4_·7H_2_O, 4 × 10^−5^ g L^−1^, and CuSO_4_·5H_2_O, 4 × 10^−5^ g L^−1^. The pH of the final solution was adjusted to 6.0 ± 0.5 [14]. After 30 days, healthy seedlings were transferred into a 192 mL glass vase (one plant per vase) containing the final solution as media in a deep-water culture system (soilless culture) Figure 4. Subsequently, sodium chloride NaCl was added at different concentrations from 50, 100, 150, and 200 mM and 0 mM (Control) 2 days after transplanting to allow acclimatization. The experiment was maintained under optimal conditions with natural light conditions of day length of 12 h and a controlled light environment of 12 h (12 h light/12 h dark photoperiod) in the greenhouse.

### 4.2. Cyanobacteria Strains Cultivation and Growth Conditions

A freshwater filamentous and N-fixing cyanobacteria namely, *Roholtiella* sp. (QUCCCM97) was selected for this study based on the enhancement potential earlier established on bell pepper (*Capsicum annuum* L.) in a previous study conducted [17]. The strain, isolated from the Qatar desert, belongs to the Qatar University Culture Collection of Cyanobacteria and Microalgae (QUCCCM) [51]. The cultivation of *Roholtiella* sp. (QUCCCM97) was performed as described by Bello et al. [17]. One single colony of the cyanobacteria strain was used to inoculate a 5 mL volume of BG11 growth medium [52]. Thereafter, incubated for 7 days at 30 °C, a photon flux density of 1.0 × 10^−4^ mol photons m^−2^ s^−1^ and a 12:12 h dark: light cycle with 150 rpm agitation using an illuminated shaker (Innova 44R, New Brunswick Scientific, Enfield, Connecticut, USA). However, the scale-up of the culture to 500 mL was gradually attained and incubated under the previously described conditions. Furthermore, an adequate volume was used to inoculate a DASGIP parallel 1L bioreactor system for phototrophic cultivation (#76DG08PBBB, Eppendorf, Hauppauge, NY, USA). This culture was grown at 30 °C, pH 8, under 300 rpm agitation to avoid settling of the cyanobacteria isolates, with 100 μmol photons m^−2^ s^−1^, a 12:12 h dark: light and 5% CO_2_ during the light phase [53]. After 15 days of incubation, the biomass from *Roholtiella* sp. (QUCCCM97) species was harvested by centrifugation then freeze-dried. All cultures were performed in duplicate [17].

### 4.3. Preparation of Cyanobacteria Extracts

The *Roholtellia* sp. freeze-dried biomass obtained after 15 days of cultivation previously described [17] was divided into two parts to maximize utilization. The first part was kept at −80 °C and the second fraction was subjected to aqueous extraction. To this end, 100 mg of dry biomass of *Roholtellia* sp. strain was first washed with sterile distilled water then dissolved into 12.5 mL phosphate buffer (0.1 M pH 6.0) before sonication for 10 min (5 s pulses of 8 W over 30 s, on ice, Sonics VCX 130 Ultrasonic processor). The phosphate buffer solution was used to stabilize and maintain the pH of the system and was not necessarily considered to have any influence on the nutrient composition of the extract and the subsequent growth-enhancing potential of the seedlings. Furthermore, extraction tubes were incubated at 4 °C for 24 h. After centrifugation at 13,000 rpm for 10 min, aqueous extracts were collected and freeze-dried. In this case, the cyanobacteria extract stock solutions were denoted as *Roholtiella* sp. QUCCCM97extr. In reality, the total time of the extraction for Roholtiella sp. QUCCCM97extr (from cell break up to the analysis of pigments) did not exceed 30 h [17].

### 4.4. Nutrient Composition of Cyanobacteria Extract Analysis

The chemical composition of *Roholtiella* sp. extract was determined by Ion Chromatography in the Central Laboratory Unit of the Qatar University, Qatar. The chemical analyzed are Sodium (Na^+^), Ammonium (NH_4_^+^), Potassium (K^+^), Calcium (Ca^2+^), Magnesium (Mg^2+^), Fluoride (F^−^), Chloride (Cl^−^), Nitrate (NO_3_^−^), Phosphate (PO43-), Sulphate (SO_4_^2−^). Also, the targeted compounds were previously determined through the spectral scan of the strain [17].

### 4.5. Experimental Design and Treatments with Microalgae Extracts

**Figure 6:**
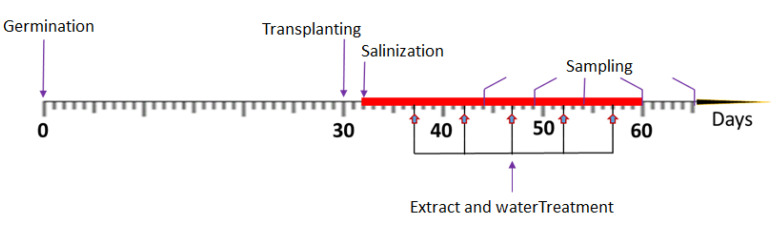
Scheme of treatment: Adapted from [54] and modified as deemed fit. Note. Salinity treatment was started on day 32 to day 60, and *Roholtiella* sp. extract and water treatment commenced at day 37 and continued at every five days intervals. Sampling was carried out on different days for the vegetative and biochemical analysis.

A factorial experiment with two factors (Foliar spraying application and salt treatments) of plant ceramic vase (one plant per vase) designed based on Completely Randomized Design (CRD) was conducted with four replications (Figure 6). Salt-stressed bell pepper (*Capsicum annuum* L.) plants were treated with microalgae extract and distilled water through foliar spraying respectively. The salt stress treatments that were applied to bell pepper seedlings (32 days old seedlings) at different concentration levels were 0, 50, 100, 150, and 200 mM of Sodium Chloride (NaCl) respectively and the solutions were checked every 3 days and refreshed if necessary. However, the sequel to the optimal cyanobacteria extracts previously reported when three strains QUCCCM97 *Roholtiella* sp., QUCCCM99 *Nostoc ellipsosporum*, QUCCCM112 *Desmonostoc danxiaense* were screened [17] Thus, the extract was tested at 6 mL L^−1^ and was sprayed at the rate of 0.576 mL/stroke/leaf and subsequently increased to two strokes as the leaves expanded. The application of the extract and water on salt-stressed seedlings was carried out for the first time 5 days after the salt-stress induction (at day 37) and the foliar extract and water application to the salt-stressed continue every 5 days interval for 28 days Figure 6. The plant treatment lasted for thirty days (after transplanting) under regulated environmental conditions at the Biological and Environmental Sciences Department greenhouse facility at the optimal conditions. After the experiment at 35 days old, the final sampling was conducted and plants were harvested, bagged with frozen bags individually, and stored at −80 °C. Prior to the harvesting, the analysis of the randomly sampled bell pepper seedlings for the different vegetative parameters viz. the shoot height, root length, number of leaves, total fresh weight, dry weight, as well as relative water content was conducted. In addition, the biochemical analysis was conducted as the pigments assay (Chlorophyll a, b, and total chlorophyll) and antioxidants assays (ABTS and catalase assay). Analysis of the total proline content in the salt-stressed (0–200 mM NaCl) plants, as well as treated plants (stress-induced 0–200 mM NaCl), was carried out after the treatments. These biochemical analyses were limited to the two most homogeneous replicates in each treatment and the leaves were randomly sampled for the different analyses. However, the entire experiments were conducted in quadruplicates and maintained at optimal conditions in the greenhouse.

### 4.6. Vegetative/Growth Characteristics

#### 4.6.1. Shoot Length (cm)—SL

Plant shoot length (cm) was measured twice, commencing ten days after transplanting and at the end of the experiment, using a steel measuring tape (STANLEY 8 m/26′, Tylon). The entire plants population was measured i.e., four plants per treatment. Thus, the average length was recorded.

#### 4.6.2. Root Length (cm)—RL

The bell pepper root length (cm) was taken at the end of the experiment using a steel metal tape (STANLEY 8 m/26′, Tylon) from the tip of the well-developed root to the point where the shoot emerges and the average value was taken.

#### 4.6.3. Number of Leaves Per Plant—NL

This measurement was taken manually by counting the number of visible leaves at 20 days of the experiment, long before the end of the experiment.

#### 4.6.4. Fresh Weight (g)—FW

The weight of the whole bell pepper plant was recorded at the termination of the experiment on the last day of the experiment with a precision weight balance and the average weight was taken.

#### 4.6.5. Dry Weight (g)—DW

The fully grown seedlings were harvested after the experiment and oven-dried (Genlab Drying Cabinet, Genlab Limited. Cheshire, UK) at 70 °C until the weight attained constancy. Subsequently, the dried samples were weighed with the precision weight and the average weight recorded for further analysis.

### 4.7. Biochemical Analyses

#### 4.7.1. Relative Water Content (RWC)

The relative water content (RWC) was determined to estimate the amount of moisture present in the leaf which was conducted by measuring five fresh leaf discs. The fresh weight (FW) of 1-cm discs [55,56] was determined with “high precision weight balance” and subsequently submerged in Petri dishes containing deionized water for 24 h and the weight was taken again to determine a complete turgid weight (TW). Subsequently, the leaf disc was oven-dried at 80 °C until the attainment of stable weight, and the final dry weight (DW) was recorded [26]. Thus, RWC was measured as follows:

$$\mathrm{RWC}=\frac{\mathrm{FW}-\mathrm{DW}}{\mathrm{TW}-\mathrm{DW}}$$

#### 4.7.2. Determination of Chlorophyll Concentrations (*N*,*N*-Dimethyl Formamide Assay)

The freshly cut bell pepper leaf discs (~100 mg) were placed in a test tube containing *N*,*N*-dimethyl formamide (DMF) [56]. Subsequently, the mixture was kept overnight (24 h) in the refrigerator at 4 °C. Thereafter, an analytical procedure was conducted with a spectrophotometer (Jenway 6715 UV/Visible Scanning Spectrophotometer, 1.5-nm Bandwidth) to determine the absorbance of the greenish supernatant at wavelengths 647 and 666 nm respectively [57].

Equations used to determine the values are shown below:

(1) $$\mathrm{Chl}\;a\;\left({\mathrm{mg}\;g}^{-1}\mathrm{fw}\right)=\frac{12.7\left(A_{664.5}\right)-2.79\left(A_{647}\right)}{1000\times W\times a}\times V$$

(2) $$\mathrm{Chl}\;b\;\left({\mathrm{mg}\;g}^{-1}\mathrm{fw}\right)=\frac{20.7\left(A_{647}\right)-4.62\left(A_{664.5}\right)}{1000\times W\times a}\times V$$

(3) $$\mathrm{Chl}\;a+b\;\left({\mathrm{mg}\;g}^{-1}\mathrm{fw}\right)=\frac{17.9\left(A_{647}\right)+8.08\left(A_{664.5}\right)}{1000\times W\times a}\times V$$

where, A = absorbance, a = length of the light path in the cell (1 cm—constant), V = volume of the extract in ml. and W = fresh weight of the sample in g.

#### 4.7.3. Proline Assay

The plant material of approximately 0.5 g was smatched/grounded in 10 mL of 3% sulfosalicylic acid which should be freshly prepared at all times. The mixture was filtered through filter paper and the residue was discarded. Thereafter, 2 milliliters of supernatant was reacted with 2 mL ninhydrin and subsequently, 2 mL of glacial acetic was added to the mixture in a glass test tube. Interestingly, the final mixture was placed in a water bath for 1 h at 90–100 °C; thereafter the mixture was submerged in ice to stop the reaction and incubated for 5 min. Furthermore, the extract was obtained from the mixture after adding 4 mL of toluene and vortex strenuously for ~20 s. The topmost layer of the mixture was collected and the absorbance was recorded spectrophotometrically (Jenway 6715 UV/Visible Scanning Spectrophotometer, 1.5-nm Bandwidth) at 520 nm wavelength with toluene as a blank [58] and proline accumulation was computed from the standard curve using the below equation as µmole g^−1^ fresh weight FW. Finally, the proline standard curve was prepared by using proline standard or L-Proline, (S)-Pyrrolidine-2-carboxylic acid.

(4) $$\mathrm{Proline}\;\mathrm{content}\;\left({µ\mathrm{moles}\;g}^{-1}\mathrm{FW}\right)=\left\{\begin{array}{c} \left({µg\;\mathrm{proline}\;\mathrm{mL}}^{-1}\times \mathrm{ml}\;\mathrm{toluene}\right)\\ /115.5 \end{array}\right\}\times \left\{\begin{array}{c} 5\\ /\left(g\;\mathrm{sample}\right) \end{array}\right\}$$

#### 4.7.4. Antioxidant/Enzymatic Activity

The antioxidant assay was carried out using two different procedures to measure antioxidant capacity and activity respectively, as one procedure might not be enough to precisely predict the antioxidant patterns. 2,2′-azino-bis (3-ethylbenzothiazoline-6-sulfonic acid) (ABTS) assay measures the antioxidant capacity and catalase assay measures the antioxidant activity respectively.

#### 4.7.5. Plant Extraction

For the investigation of catalase, 500 mg of fresh leaf tissue was frozen in liquid nitrogen, subsequently ground in a 5 mL extraction buffer prepared from 0.1 M phosphate buffer (7–7.5 ph), 0.0005 M EDTA, and 0.1% polyvinyl pyridine (PVP) respectively. The mixture was centrifuged at 15,000× *g* for 20 min at 4 °C. The collected supernatant was used for different assays as enumerated below. The temperature of enzyme preparation and activity was maintained at 4 °C throughout. The protein content was determined from the aliquot obtained from the extract by using the Bradford method (Bradford, 1976) while the standard curve was generated from bovine serum albumin (BSA).

#### 4.7.6. 2,2′ Azinobis (3-Ethylbenzothiazoline-6-Sulphonic Acid) (ABTS) Assay

The concept of ABTS assay is the production of ferryl myoglobin radical from metmyoglobin and H_2_O_2_; thus, it lead to the oxidation of ABTS to form radical cations ABTS^+^, a greenish chromagen with high solubility potential and is measurable spectrophotometrically at a wavelength of 405 nM. The total antioxidant capacity of leaf tissues was determined instantly at the expiration of the exposure period. Fresh leaves were collected randomly from the four replicates as designed. The description and procedure explained in the assay kit (Antioxidant assay CS0790, Sigma-Aldrich Co. LLC. St. Louis, Missouri, USA) were followed to carry out the extraction of the enzyme and subsequently, the capacity measurement. Summarily, ~100 mg leaf tissue was frozen in liquid nitrogen, grinded, and subsequently homogenized in 0.5 mL 1× Assay Buffer, then centrifuged at 12,000× *g* at 4 °C for 15 min. Thereafter, the assay was conducted/carried out/prepared in the 96 well plates. The first 1–12 wells contained the synthetic Trolox employed to generate the standard curve that was subsequently used for antioxidant activities quantification, 10 µL of a Trolox standard solution, and 20 µL of Myoglobin working solution were added. Subsequently, in the wells for the test samples 1–12 wells, 10 µL of leaf tissue samples (supernatant—~100 mg/0.5 mL of 1× Assay Buffer) and 20 µL of Myoglobin were added. To each well, 150 µL of ABTS substrate working solution (addition of 25 µL of Hydrogen Peroxide; H_2_O_2_ to 10 µL of ABTS Substrate Solution/Master solution) was added. The homogenates were incubated for 5 min at room temperature and thereafter, 100 µL stop solution was added to each well to terminate the reaction. However, before adding the stop solution, it was warmed to room temperature and mixed thoroughly until homogeneous. The plate reader was used to read the endpoint absorbance at 405 nm wavelength. The antioxidant capacity results were revealed as coequal of mM Trolox Equivalent (TE) per g of fresh weight of the samples (mM of TE g^−1^ FW) using the below Equation (5).

(5) $$Z\left(\mathrm{mM}\right)=\frac{y\;\left(A_{405}\right)-\mathrm{Intercept}}{\mathrm{Slope}}\times \;\mathrm{dilution}\;\mathrm{factor}$$

where, Z (mM)—Antioxidant concentration [(mM) relative to the Trolox standard curve concentration], y(A_405_)—the average absorbance of the leaf tissue samples at 405 nm, Intercept—stands for the intercept of the Y-axis by the standard curve, the dilution factor is used only if the sample should be diluted before adding to the well. It is the fold dilution of the original sample.

#### 4.7.7. Catalase Activity Assay

The activity of catalase was measured and estimated according to Aebi et al., 1984 [54,59]. The constituent of the reaction mixture contained 0.1 mL enzyme extract, 9.9. mL 0.1 M phosphate buffer (pH 7.0) and 0.5 mL 0.03 M H_2_O_2_ adding up to final volume of 1.5 mL. The addition of H_2_O_2_ occurs lastly and absorbance was measured spectrophotometrically at a wavelength of 240 nm. The enzymatic breakdown and subsequent disappearance of the substrate (H_2_O_2_) were monitored as absorbance decreased for 30 min as further illustrated in the Equation (6).

(6) $$H_{2}O_{2}\;\overset{\mathrm{catalase}}{\to }\;2H_{2}O+{\;O}_{2}$$

Catalytic activity to decompose H_2_O_2_ to produce H_2_O and O_2_ respectively.

While the computation of the catalase activity is shown in Equation (7).

(7) $$\mathrm{Catalase}\;\mathrm{activity}\;\left({\mathrm{unit}\;\mathrm{mg}\;\mathrm{protein}}^{-1}{\;\min}^{-1}\right)=\Delta A_{240}\left(1000{|\varepsilon }_{I}*\mathrm{PC}\right)$$

where ∆A_240_ is the change in catalase absorbance at 240 wavelengths, **ε_i_** is extinction coefficient (40 mM^−1^ cm^−1^) for H_2_O_2_ [60], and PC is the protein content respectively.

### 4.8. Statistical Analysis

The statistical data analysis was determined by the analysis of variance (two-way ANOVA) using a Minitab version 19. The data reported are mean values ± SD. The mean comparison of values was conducted by Tukey’s post hoc test of Pairwise Comparisons while differences were considered significant at *p*-Value less than 0.05 (*p* < 0.05) as represented with different letters.

## 5. Conclusions

In the present study, we can conclude that bell pepper (*Capsicum annuum* L.) induced with salt stress at various concentrations (50, 100, 150, and 200 mM) was negatively affected and this impact can be minimized by foliar application of *Roholtiella* sp. extract. The extract was advantageous and played a crucial role in attenuating the adverse impacts of salt stress on bell pepper vegetative growth, biochemical characteristics, and enzymatic activity. The application of *Roholtiella* sp. extract resulted in an increased shoot length, root length, number of leaves, fresh weight, dry weight, chlorophyll content, proline accumulation, and enzymatic activity. However, from this study, the obtained results will support the enhancement of bell pepper production under salt stress in large-scale field and hydroponic production systems by the application of *Roholtiella* sp. extract treatments. Interestingly, to the best of our understanding, this report on the application of cyanobacteria (*Roholtiella* sp.) high-value product extract in salt stress mitigation on sweet pepper (*Capsicum annum* L.) is likely the first study of its kind in Qatar and probably in the entire Gulf Cooperation Council (GCC) countries.

## Data Availability

Not applicable.
